# Cystatin C based estimation of chronic kidney disease and amyotrophic lateral sclerosis in the ALS registry Swabia: associated risk and prognostic value

**DOI:** 10.1038/s41598-023-46179-9

**Published:** 2023-11-10

**Authors:** Gabriele Nagel, Deborah Kurz, Raphael S. Peter, Angela Rosenbohm, Wolfgang Koenig, Luc Dupuis, Hansjörg Bäzner, Axel Börtlein, Silke Dempewolf, Martin Schabet, Martin Hecht, Andreas Kohler, Christian Opherk, Andrea Naegele, Norbert Sommer, Alfred Lindner, Hayrettin Tumani, Albert C. Ludolph, Dietrich Rothenbacher

**Affiliations:** 1 Institute of Epidemiology and Medical Biometry, Ulm University, Helmholtzstr. 22, 89081 Ulm, Germany; 2 Department of Neurology, Ulm University, Ulm, Germany; 3 Deutsches Herzzentrum München, Technische Universität München, Munich, Germany; 4 DZHK (German Centre for Cardiovascular Research), Partner Site Munich Heart Alliance, Munich, Germany; 5 Université de Strasbourg, Inserm, UMR-S1118, Centre de Recherches en Biomédecine de Strasbourg, Strasbourg, France; 6 Department of Neurology, Klinikum Stuttgart, Stuttgart, Germany; 7 Department of Neurology, RKH Klinikum Ludwigsburg, Ludwigsburg, Germany; 8 Department of Neurology, Klinikum Kaufbeuren, Kliniken Ostallgäu Kaufbeuren, Kaufbeuren, Germany; 9 Department of Neurology, Klinikum am Gesundbrunnen Heilbronn, Heilbronn, Germany; 10 Department of Neurology, Christophsbad Goeppingen, Goeppingen, Germany; 11 Department of Neurology, Marienhospital Stuttgart, Stuttgart, Germany; 12 Deutsches Zentrum Für Neurodegenerative Erkrankungen (DZNE), Ulm, Germany

**Keywords:** Amyotrophic lateral sclerosis, Predictive markers, Prognostic markers

## Abstract

Kidney function as part of metabolic changes could be associated with amyotrophic lateral-sclerosis (ALS). We investigated the associations between estimated chronic kidney disease (CKD), based on the Chronic Kidney Disease Epidemiology Collaboration (CKD-EPI) cystatin C equation, and the risk at onset and prognostic value of CKD for ALS. Between October 2010 and June 2014, 362 ALS cases (59.4% men, mean age 65.7 years) and 681 controls (59.5% men, means age 66.3 years) were included in a population-based case–control study based on the ALS registry Swabia in Southern Germany. All ALS cases were followed-up (median 89.7 months), 317 died. Serum samples were measured for cystatin C to estimate the glomerular filtration rate (eGFR) according to the CKD-EPI equation. Information on covariates were assessed by an interview-based standardized questionnaire. Conditional logistic regression models were applied to calculate odds ratios (OR) for risk of ALS associated with eGFR/CKD stages. Time-to-death associated with renal parameters at baseline was assessed in ALS cases only. ALS cases were characterized by lower body mass index, slightly lower smoking prevalence, more intense occupational work and lower education than controls. Median serum cystatin-C based eGFR concentrations were lower in ALS cases than in controls (54.0 vs. 59.5 mL/min pro 1.73 m^2^). The prevalence of CKD stage ≥ 3 was slightly higher in ALS cases than in controls (14.1 vs. 11.0%). In the adjusted models, CKD stage 2 (OR 1.82, 95% CI 1.32, 2.52) and stage 3 (OR 2.34, 95% CI 1.38, 3.96) were associated with increased ALS risk. In this cohort of ALS cases, eGFR and CKD stage ≥ 3 (HR 0.94; 95% CI 0.64, 1.38) were not associated with prognosis. In this case–control study, higher CKD stages were associated with increased ALS risk, while in the prospective cohort of ALS cases, no indication of an association of CysC-based CKD on mortality was seen. In addition, our work strengthens the importance to evaluate renal function using a marker independent of muscle mass in ALS patients.

## Introduction

Amyotrophic lateral sclerosis (ALS) is a rare neurodegenerative disease affecting progressively different parts of the body and going along with progressive motor neuron degeneration. The mean survival in ALS-patients is about 3–4 years. The precise aetiology and biological mechanisms underlying ALS remain unclear. During recent years, multiple factors contributing to the development and progression of ALS have been suggested^1^. Metabolic alterations were observed in patients with ALS and in animal models contributing to the evidence that dysregulation in energy metabolism is associated with ALS risk^2–4^.

Metabolic alterations appear causally linked to ALS disease progression as shown by case-control studies focusing on weight loss^5–8^, metabolic hormones^9–11^, or inverse association with diabetes mellitus^12^. From a broader perspective, ALS appears generally associated with physical fitness^13^ and decreased incidence of chronic diseases^14^.

Among chronic diseases possibly associated with ALS, kidney disease appears particularly interesting and under-studied. Indeed, Mitchell et al.^14^ found a decreased risk association between prior kidney disease and ALS in an observational study, while in another study, existing kidney disease was associated with shorter survival^15^. The lack of information on possible relationships between ALS and kidney function is likely due to the fact that serum creatinine is generally used as a biomarker of kidney function in the general population. However, serum creatinine is heavily confounded in ALS patients by decreased muscle mass and venous creatinine actually correlates with fat free mass in patients with ALS^16^.

In clinical situations with secondary muscle mass loss or neurological diseases^17^, it is generally recommended to evaluate kidney function using cystatin C-based equations. Cystatin C (CysC) is a lysosomal protein acting as an endogenous inhibitor of cathepsins^18^. CysC is also extensively expressed in neurons, astrocytes, endothelial and microglial cells in the brain and is also found in body fluids. Importantly, CysC is unaffected by several factors such as age, sex, and muscle mass^19^ and CysC concentrations were found to be a reliable marker for kidney function overall^20^. CysC based equations outperformed creatinine equations in evaluating kidney function in primary neuromuscular diseases^21^ and in ALS patients^22^.

To date, little is known about the association of (chronic kidney disease) CKD with ALS onset and progression. In order to clarify the relation between kidney function and risk of ALS without the confounding effect of decreased muscle mass, we measured CysC concentrations and applied the Chronic Kidney Disease Epidemiology Collaboration (CKD-EPI) equation, based on CysC in a registry-embedded case–control study^20^. In addition, we used the prospective data of the ALS cases to investigate the prognostic value of cystatin- C based kidney function according to CKD-Epi stages in ALS with respect to overall survival.

## Material and methods

### Study design and study population

The ALS registry Swabia has been described previously in detail^23–25^. In brief, it is a population-based clinical-epidemiological registry with the aim to collect data on all newly diagnosed ALS cases in Swabia, a defined geographic region with approximately 8.4 million inhabitants in the South-West of Germany.

All reported ALS cases were defined by the diagnosis of possible, probable or definite ALS according to the revised El Escorial criteria by an experienced neurologist^26^. Notifications of patients with suspected ALS were re-evaluated during the clinical course by the registry.

Patients registered between October 01, 2010 and June 30, 2014 were offered to provide informed consent to participate in a population-based case–control study. For each case (N = 362), two sex and age frequency-matched control subjects (N = 681) were randomly selected from the general population as registered in the regional registry office (“Einwohnermeldeamt”). The identified subjects were contacted by postal mail and invited to participate in the study. After written informed consent was obtained, study nurses visited the participants for a standardized interview and blood sampling. The standardized instruments and tests were performed identically in ALS cases and controls. Information on body mass index (BMI), smoking status, educational attainment, family history of ALS, occupation work intensity and medical history of hypertension, diabetes mellitus, chronic heart disease, and myocardial infarction was collected^24^. Response in cases was 65% (20% refused and 15% could not be contacted) and in the population-based controls 19% (39% refused and 42% did not respond after several attempts to get in contact per mail and telephone).

In addition, ALS cases were actively followed-up annually and interviewed. To update vital status record linkage with the central registration offices in Baden-Württemberg and Bavaria were performed (last update December, 2020). In case of death, the date of death was obtained from the local registration office.

### Ethics statement

All methods were carried out according to international, national, and state rules in implementing the ALS registry in Swabia. We obtained approval of the ethical committees of Ulm University and the regional physician chambers ("Landesaerztekammer Baden-Wuerttemberg" and "Landesaerztekammer Bayern").

### Laboratory measurements

According to a common standard protocol for cases and controls, blood samples were transported in cooled containers to the study center. Serum was obtained by centrifugation for 10 min at 2000 RPM×*g* and 4 °C (Heraeus Multifuge 3 S-R, Fa. Thermofischer). Blood specimens were transferred into 0.5–1.0 mL aliquots with screw tops on the same day and stored at − 80° Celsius until further analysis. CysC (mg/L) was measured in serum using an immunonephelometric assay [N Latex Cystatin C, 2010; Siemens, Eschborn, Germany; LOD 0.05 mg/L, measuring range 0.05–7.25 mg/L, intra-assay CV 2.3%, inter-assay CV 2.9–3.2%]. All laboratory analyses were performed in blinded fashion at the Biomarker Laboratory of the Department of Internal Medicine II-Cardiology, Ulm University Medical Center.

### Statistical methods

Conditional logistic regression was used to calculate multivariable odds ratios (OR) and 95% confidence intervals (CI) for the association of renal function (i.e. stages of CKD and ALS. Glomerular filtration rate (eGFR) was calculated based on the CKD-EPI CysC equation^27^ CKD stages were categorized according to cut-points suggested by Inker et al.^28^ Adjustment variables were identified by Directed Acyclic Graphs (DAG)^29^. Models were conditioned on age and sex, and adjusted for BMI, diabetes mellitus, and smoking (ever) (details see Table 2). The results of the alternative minimal sufficient adjustment replacing smoking by cardiovascular disease (CVD¸history of either hypertension or/and, coronary heart disease or/and, myocardial infarction or/and, stroke) is presented in the Supplement.

The Cox proportional hazards model was adjusted for age and sex. Additional adjustment for diagnostic delay, site of onset, ALS-functional risk score (FRS), body mass index, self-reported diabetes and smoking (ever) status were applied to calculate hazard ratios (HRs) for overall survival in ALS cases only [Model 4a adjusted: age, sex, diagnostic delay, site of onset, ALS-FRS, Model 4c adjusted: age and sex, diagnostic delay, site of onset, ALS-FRS, body mass index, self-reported diabetes and smoking (ever)]. Survival times were calculated until date of death, date of tracheostomy with invasive ventilation (TIV) or date of the last systematic mortality update (December, 2020), whatever came first. The proportional hazards assumption was assessed graphically. Sensitivity analyses excluding the El Escorial categories "clinically suspected" and "clinically possible" were performed in the adjusted model. All provided p-values are two-sided. The statistical software package SAS release 9.4 (SAS Institute, Cary, NC, USA) was used.

## Results

In the case–control study data from 362 cases (mean age 65.7 (SD 10.6) years, 59.4% male) and 681 controls (mean age 66.3 (SD 9.9) years, 59.5% male) were included (Table 1). ALS cases were characterized by lower mean BMI (24.5 (SD 4.0) vs. 26.5 (SD 4.1) kg/m^2^), slightly lower smoking prevalence (ever: 48.7% vs. 49.2%), more intense occupational work (physically demanding: 22.0% vs. 12.5%) and lower education (≥ 10th grade: 44.8% vs. 56.1%) than controls. ALS onset was lumbar (n = 120 [33.2%]), bulbar (n = 116 [32.0%]), or cervical (n = 106 [29.3%]). Based on the revised El Escorial criteria, more than 70% of ALS cases had a probable or definite clinical diagnosis.

**Table 1:** Main characteristics of ALS cases and control subjects.

Case–control study	N_Cases_	ALS-cases	N_Controls_	Control subjects
	362		681	
Age (years), mean (SD)	362	65.7 (10.6)	681	66.3 (9.9)
Age (years), N (%)	362		681	
< 65		159 (43.9)		273 (40.1)
≥ 65		203 (56.1)		408 (59.9)
Sex	362		681	
Male, N (%)		215 (59.4)		405 (59.5)
Family history of ALS, N (%)	356		681	
Positive		15 (4.2)		4 (0.6)
School education, N (%)	362		678	
< 10th grade (1)		200 (55.3)		298 (44.0)
≥ 10th grade (0)		162 (44.8)		380 (56.1)
Smoking	357		679	
Ever, N (%)		174 (48.7)		334 (49.2)
BMI (kg/m^2^), mean (SD)	362	24.5 (4.0)	679	26.5 (4.1)
< 23 kg/m^2^, N (%)		136 (37.6)		126 (18.6)
23–< 25 kg/m^2^, N (%)		80 (22.1)		146 (21.5)
25–< 28 kg/m^2^, N (%)		79 (21.8)		201 (29.6)
≥ 28 kg/m^2^, N (%)		67 (18.5)		206 (30.3)
Occupational work intensity, N (%)	350		675	
Light (mainly sitting)		124 (35.4)		325 (48.2)
Moderate (standing and walking)		149 (42.6)		266 (39.4)
Heavy (physically demanding)		77 (22.0)		84 (12.5)
History of, N (%)				
Hypertension	351	170 (48.4)	671	312 (46.5)
Diabetes mellitus	353	37 (10.5)	667	70 (10.5)
Cardiovascular diseases^a^	356	183 (51.4)	677	336 (49.6)
Chronic kidney disease	239	3 (1.3)	552	16 (2.9)
Cystatin C (mg/L), median (Q1, Q3)	362	0.91 (0.81, 1.00)	681	0.88 (0.78, 1.01)
eGFR (CKD-EPI), median (Q1, Q3)	362	83.1 (69.1, 98.8)	681	86.4 (72.6, 100.5)
CKD stage ≥ 3 (< 60 mL/min 1.73^–2^ m^−2^), N (%)	362	51 (14.1)	681	75 (11.0)
CKD-stage 1 (eGFR ≥ 90)	362	141 (39.0)	681	312 (45.8)
CKD-stage 2 (≥ 60 eGFR < 90)		170 (47.0)		294 (43.2)
CKD-stage 3 (≥ 30 eGFR < 60)		47 (13.0)		64 (9.4)
CKD-stage 4 (≥ 15 EGFR < 30)		4 (1.1)		7 (1.0)
CKD-stage 5 (EGFR < 15)		0 (0.0)		4 (0.6)
Clinical characteristics of ALS-cases				
Site of onset, N (%)	362			
Bulbar		116 (32.0)		
Cervical		106 (29.3)		
Thoracic		14 (3.9)		
Lumbar		120 (33.2)		
Uncertain		6 (1.7)		
Revised El Escorial criteria, N (%)	362			
Clinically suspected		70 (19.3)		
Clinically possible		38 (10.5)		
Clinically probable		224 (61.9)		
Clinically definite		30 (8.3)		

^a^history of cardiovascular diseases = history of either hypertension or/and, coronary heart disease or/and, myocardial infarction or/and, stroke.

*eGFR* estimated glomerular filtration rate, per mL/min/1.73 m^2^, *CKD* chronic kidney disease, *BMI* body mass index.

Clinically suspected ALS at baseline progressed to advanced stages of El Escorial criteria during observation.

The median serum CysC concentration was numerically higher in ALS cases than in controls (0.91 vs. 0.88 mg/L). Concerning renal function, the eGFR was lower in ALS cases than in controls (83.1 vs. 86.4 mL/min/1.73 m^2^), while the prevalence of CKD stage 3 and more was higher in ALS cases than controls (in sum 14.1 vs. 11.0%). Compared to controls, ALS cases had higher prevalence of hypertension (48.4 vs. 46.5%) and CVD (51.4 vs 49.6%), while self-reported CKD (1.3 vs. 2.9%) was less prevalent, and diabetes mellitus (10.5 vs. 10.5%) was identically reported.

In the case–control study, eGFR as a continuous variable was inversely associated with ALS risk in the model adjusted for age, sex, BMI, self-reported diabetes and smoking (ever) (per 10 units increase: OR 0.86, 95% CI 0.80, 0.94) (Table 2). CKD stages ≥ 3 vs. stages 1 and 2 were associated with increased risk of ALS (OR 1.39 95% CI 0.88, 2.19), but the association was not statistically significant. When looking at single stages with reference group CKD-stage 1, CKD stage 2 (OR 1.82, 95% CI 1.32, 2.52) and stage 3 (OR 2.34, 95% CI 1.38, 3.96) were positively associated with ALS risk in the adjusted models. Overall, CKD stages were also associated with increased ALS risk (p for trend 0.0024). The alternative minimal sufficient adjustment set, including CVD, revealed slightly weaker associations (Supplement Table S1). However, the pattern remained similar for CKD stages (p for trend 0.0099).

**Table 2:** Odds ratios for ALS associated with eGFR/CKD-stages.

	eGFR/CKD-stages odds ratio (95%-CI)
eGFR	
Adjusted a (N_Cases_ = 353, N_Controls_ = 665)^a^	
Per 10 units increase	0.90 (0.83, 0.97)
p-value	0.0047
Adjusted b (N_Cases_ = 348, N_Controls_ = 663)^b^	
Per 10 units increase	0.86 (0.80, 0.94)
p-value	0.0005
CKD stage ≥ 3 (eGFR < 60)	
Adjusted b (N_Cases_ = 348, N_Controls_ = 663)^b^	
CKD-stage 1&2	(ref.) 1.00
CKD-stage ≥ 3	1.39 (0.88, 2.19)
p-value	0.16
CKD-stages	
Adjusted b (N_Cases_ = 348, N_Controls_ = 663)^b^	
CKD-stage 1	(ref.) 1.00
CKD-stage 2	1.82 (1.32, 2.52)
CKD-stage 3	2.34 (1.38, 3.96)
CKD-stage 4	1.66 (0.43, 6.38)
CKD-stage 5	–
p-value for trend^c^	0.0024

^a^Conditioned age and sex.

^b^As in footnote a, but additionally adjusted for BMI, self-reported diabetes, smoking (ever).

^c^p-value for trend over stages.

*eGFR* estimated glomerular filtration rate, per mL/min/1.73 m^2^, *CKD* chronic kidney disease.

317 (88%) of 362 ALS cases, died during a median follow-up of 89.7 months. Compared to the deceased, survivors were characterized by younger mean age (62.6 vs. 66.2 years), were more frequently male (77.8% vs. 56.8%), had a higher a mean BMI (25.3 vs. 24.4 kg/m^2^), longer median diagnostic delay (7.0 vs. 5.0 months) and higher median ALS-FRS (43 vs. 38 points) (Table 3). Median eGFR were higher among survivors (85.0 vs. 81.9 mL/min/1.73 m^2^). Survivors were characterized by lower CKD stages.

**Table 3:** Characteristics of the cohort of ALS cases (N = 362) by survival status.

	N_Deceased_	Deceased	N_Survived_	Survived
Age (years), mean (SD)	317	66.2 (10.6)	45	62.6 (10.2)
Sex	317		45	
Male, N (%)		180 (56.8)		35 (77.8)
BMI (kg/m^2^), mean (SD)	317	24.4 (4.0)	45	25.3 (4.3)
Diagnostic delay (month), median (Q1, Q3)	317	5.0 (2.9, 9.0)	45	7.0 (2.0, 12.0)
ALS-FRS, median (Q1, Q3)	316	38 (33, 42)	45	43 (39, 45)
Cystatin C (mg/L), median (Q1, Q3)	317	0.91 (0.81, 1.04)	45	0.92 (0.81, 1.03)
eGFR, (CKD-EPI equation), mean (SD)	317	81.9 (20.7)	45	85.0 (17.3)
CKD-stages, N (%)				
CKD-stage 1 (eGFR ≥ 90)	317	125 (39.4)	45	16 (35.6)
CKD-stage 2 (≥ 60 eGFR < 90)		144 (45.4)		26 (57.8)
CKD-stage 3 (≥ 30 eGFR < 60)		44 (13.9)		3 (6.7)
CKD-stage 4 (≥ 15 egFR < 30)		4 (1.3)		0 (0.0)
CKD-stage 5 (eGFR < 15)		0 (0.0)		0 (0.0)

BMI body mass index, Q1 first quartile, Q3 third quartile.

*eGFR* estimated glomerular filtration rate, per mL/min/1.73 m^2^, *CKD* chronic kidney disease.

In model 4b adjusted for age, sex, and diagnostic delay, site of onset, and ALS-FRS, no statistically significant association was found between the increase of eGFR and the prognosis of ALS (per 10 units increase: HR 1.04; 95% CI 0.96, 1.12) and the model 4c further adjusted for body mass index, self-reported diabetes and smoking (ever) (per 10 units increase: HR 1.03; 95% CI 0.95, 1.11) (Table 4). Concerning kidney function, CKD stage ≥ 3 in the adjusted model (HR 0.94; 95% CI 0.64, 1.38) was not associated with mortality compared to CKD stage 1 and 2. When analyzing the CKD stages, for the CKD stage 4, an HR of 1.68 was found, however, the due to small numbers the confidence interval was wide and not statistically significant (95% CI 0.59–4.75). Adjustment for CVD reveal similar results for mortality by CKD stages (Table S2).

**Table 4:** Hazard ratios by eGFR/CKD-Stages among ALS cases (n = 362).

	Hazard ratio (95%-CI)
eGFR	
Crude (N_Deceased_ = 317, N_Survived_ = 45)^a^	
Per 10 units increase	0.97 (0.90, 1.03)
Per 20 units increase	0.93 (0.82, 1.07)
Per 25 units increase	0.92 (0.78, 1.09)
Per 30 units increase	0.90 (0.74, 1.10)
p-value	0.31
Adjusted b (N_Deceased_ = 316, _Survived_ = 45)^b^	
Per 10 units increase	1.04 (0.96, 1.12)
Per 30 units increase	1.09 (0.86, 1.38)
p-value	0.35
Adjusted c (N_Deceased_ = 303, N_Survived_ = 44)^c^	
Per 10 units increase	1.03 (0.95, 1.11)
p-value	0.46
CKD stage ≥ 3 (eGFR < 60)	
Adjusted c (N_Deceased_ = 303, N_Survived_ = 44)^c^	
CKD-stage 1 & 2	(ref.) 1.00
CKD stage ≥ 3 (eGFR < 60)	0.94 (0.64, 1.38)
CKD-stages	
Adjusted c (N_Deceased_ = 303, N_Survived_ = 44)^c^	
CKD-stage 1	(ref.) 1.00
CKD-stage 2	0.79 (0.59, 1.04)
CKD-stage 3	0.71 (0.44, 1.14)
CKD-stage 4	1.68 (0.59, 4.75)
CKD-stage 5	–
p-value for trend^d^	0.22

^a^Adjusted for age and sex.

^b^Additionally adjusted for diagnostic delay, site of onset, and ALS-FRS.

^c^As b, but additionally adjusted for body mass index, self-reported diabetes and smoking (ever).

^d^p-value for trend over stages.

*eGFR* estimated glomerular filtration rate, per mL/min/1.73 m^2^, *CKD* chronic kidney disease.

## Discussion

The study was conducted within the population-based ALS registry Swabia, which started to prospectively recruit newly diagnosed ALS cases in the South-West of Germany in October 2010. In the case–control study a slightly higher prevalence of CKD stages ≥ 3 was evident in ALS-cases compared to controls. In the models however, after adjustment for covariates an inverse association between eGFR with ALS risk was found. Concerning kidney function, CKD stage 3 more than doubled the ALS risk when compared to stage 1 as reference group. In the cohort of ALS cases, no clear associations of eGFR and CKD stages with all-cause mortality were observed, indicating that CKD has no prognostic value in ALS.

In the present study, we found a higher prevalence of CKD according to biomarker measurements of renal function among ALS cases than among controls (notably, CKD is rarely occurring when evaluated by self-report, respectively, hardly diagnosed in routine assessment). In contrast, Mitchell et al.^14^ reported a lower prevalence of kidney disease as a comorbid condition among patients with ALS than in the control population. The differences could be related to differences in the study population (clinical vs. population-based), the disease definition (self-reported disease evaluated by standardized questionnaire vs. biomarker measurement) and the mean age of the study samples, which was about five years higher in our population. Our findings concerning increased ALS risk among patients with decreasing eGFR and increasing CKD stages are consistent with previous observations concerning age of onset and duration of ALS^15^. In their case–control study, previous kidney disease was associated with the duration of ALS disease^15^. Yet, how CKD might influence ALS prognosis still remains to be investigated.

Tetsuka et al.^22^ characterized renal function in 76 ALS patients and 30 controls based on both creatinine and CysC-based eGFR. There findings of no association between CysC-based eGFR-measurements and ALS are in contrast with our results for low CKD stages and previous studies suggesting a utility of CysC levels in ALS diagnosis^30–32^ or prognosis^30,33^. Other studies also did not find altered levels of CysC in ALS patients neither in blood nor in cerebrospinal fluid (CSF)^34^. A meta-analysis of these studies suggested decreased levels of CysC in CSF, but unchanged levels in the blood^35^, consistent with our results. Indeed, our results do not exclude that CysC, as an inhibitor of lysosomal cathepsins could have a translational potential through a CNS local effect, as was suggested in a mouse model of ALS^36^.

While measuring renal function using CysC did not unambiguously relate ALS and CKD, levels of creatinine, another widely clinically used marker of renal function, however correlate with disease progression^37^. Critically, creatinine levels are confounded by loss of muscle mass in ALS. Indeed, Holdom and collaborators investigated the association of creatinine with disease progression^16^ and showed that blood creatinine concentration decreased with fat-free mass during progression^16^. Taken together, the previously observed relations between creatinine and disease progression are likely to be unrelated to kidney function. In all, the evidence available^16^ strengthens the importance to evaluate renal function using a marker independent of muscle mass in ALS patients, such as CysC-based eGFR^20^.

In addition, since serum creatinine levels appear negatively associated with ALS progression as a surrogate of decreased muscle mass rather than kidney function, and since moderate physical activity appears prognostically favorable^38^, maintaining muscle mass may be associated with better prognosis.

Some limitations need to be considered when interpreting the results of our study. Residual confounding cannot be ruled out. Therefore, for the case–control study part, no causal conclusion can be drawn. When generalizing the results of the case–control study, the low participation rate among controls should be considered. Due to the matched study design agegroup, sex and regional distribution were controlled. However, in the control group were more persons with higher school education (≥ 10th grade: 56.1% vs. 44.8%) and less intensive occupational work (physically demanding: 12.5% vs. 22.0%). Though we carefully used multivariable analysis in order to further adjust for potential confounder, the self selection of the control subjects may have resulted in differential estimates. CKD as a prognostic factor was analyzed in a cohort of ALS patients, who are representative for the ALS registry Swabia^24^ with a median follow-up of 89.7 months. Strengths of our study are the population-based approach and the embedded case–control study with virtually complete follow-up of ALS cases. The phenotype distribution of ALS cases recruited in the case–control study was similar to the distribution in the epidemiological ALS registry Swabia Rosenbohm et al. (2017), suggesting little selection bias^25^.

Taken together, we found evidence for an association between CKD stages with ALS risk. However, in the cohort of ALS cases, no prognostic impact of CysC -based CKD on mortality was seen.

### Supplementary Information

Supplementary Information 1.

Supplementary Information 2.

## Data Availability

Due to ethical restrictions regarding data protection issues and the study specific consent text and procedure, the data cannot be made publicly available, but the data are available from the corresponding author on reasonable request.

## References

[1] Feldman EL (2022). Amyotrophic lateral sclerosis. Lancet.

[2] Dupuis L, Pradat P-F, Ludolph AC, Loeffler J-P (2011). Energy metabolism in amyotrophic lateral sclerosis. Lancet Neurol..

[3] Guillot SJ, Bolborea M, Dupuis L (2021). Dysregulation of energy homeostasis in amyotrophic lateral sclerosis. Curr. Opin. Neurol..

[4] Nelson AT, Trotti D (2022). Altered bioenergetics and metabolic homeostasis in amyotrophic lateral sclerosis. Neurotherapeutics.

[5] Peter RS (2017). Life course body mass index and risk and prognosis of amyotrophic lateral sclerosis: Results from the ALS registry Swabia. Eur. J. Epidemiol..

[6] Westeneng H-J (2021). Associations between lifestyle and amyotrophic lateral sclerosis stratified by C9orf72 genotype: A longitudinal, population-based, case-control study. Lancet Neurol..

[7] Diekmann K (2020). Impact of comorbidities and co-medication on disease onset and progression in a large German ALS patient group. J. Neurol..

[8] Mariosa D (2017). Body mass index and amyotrophic lateral sclerosis: A study of US military veterans. Am. J. Epidemiol..

[9] Nagel G (2017). Adipokines, C-reactive protein and amyotrophic lateral sclerosis: Results from a population- based ALS registry in Germany. Sci. Rep..

[10] Nagel G (2020). Association of insulin-like growth factor 1 concentrations with risk for and prognosis of amyotrophic lateral sclerosis: Results from the ALS Registry Swabia. Sci. Rep..

[11] Rosenbohm A (2018). Association of serum retinol-binding protein 4 concentration with risk for and prognosis of amyotrophic lateral sclerosis. JAMA Neurol..

[12] Vasta R, D’Ovidio F, Logroscino G, Chiò A (2021). The links between diabetes mellitus and amyotrophic lateral sclerosis. Neurol. Sci..

[13] Mattsson P, Lönnstedt I, Nygren I, Askmark H (2012). Physical fitness, but not muscle strength, is a risk factor for death in amyotrophic lateral sclerosis at an early age. J. Neurol. Neurosurg. Psychiatry.

[14] Mitchell CS (2015). Antecedent disease is less prevalent in amyotrophic lateral sclerosis. Neurodegener. Dis..

[15] Hollinger SK, Okosun IS, Mitchell CS (2016). Antecedent disease and amyotrophic lateral sclerosis: What is protecting whom?. Front. Neurol..

[16] Holdom CJ (2021). Venous creatinine as a biomarker for loss of fat-free mass and disease progression in patients with amyotrophic lateral sclerosis. Eur. J. Neurol..

[17] Teaford HR, Barreto JN, Vollmer KJ, Rule AD, Barreto EF (2020). Cystatin C: A primer for pharmacists. Pharmacy.

[18] Gauthier S, Kaur G, Mi W, Tizon B, Levy E (2011). Protective mechanisms by cystatin C in neurodegenerative diseases. Front. Biosci..

[19] Newman DJ (2002). Cystatin C. Ann. Clin. Biochem..

[20] Shlipak MG, Coresh J, Gansevoort RT (2013). Cystatin C versus creatinine for kidney function-based risk. N. Engl. J. Med..

[21] Aldenbratt A, Lindberg C, Johannesson E, Hammarsten O, Svensson MK (2022). Estimation of kidney function in patients with primary neuromuscular diseases: Is serum cystatin C a better marker of kidney function than creatinine?. J. Nephrol..

[22] Tetsuka S, Morita M, Ikeguchi K, Nakano I (2013). Utility of cystatin C for renal function in amyotrophic lateral sclerosis. Acta Neurol. Scand..

[23] Nagel G (2013). Implementation of a population-based epidemiological rare disease registry: Study protocol of the amyotrophic lateral sclerosis (ALS)–registry Swabia. BMC Neurol..

[24] Uenal H (2014). Incidence and geographical variation of amyotrophic lateral sclerosis (ALS) in Southern Germany–completeness of the ALS registry Swabia. PLoS ONE.

[25] Rosenbohm A (2017). Epidemiology of amyotrophic lateral sclerosis in Southern Germany. J. Neurol..

[26] Brooks BR, Miller RG, Swash M, El Munsat TL, World Federation of Neurology Research Group on Motor Neuron Diseases (2000). Escorial revisited: Revised criteria for the diagnosis of amyotrophic lateral sclerosis. Amyotroph Lateral Scler. Other Motor. Neuron. Disord..

[27] Inker LA (2012). Estimating glomerular filtration rate from serum creatinine and cystatin C. N. Engl. J. Med..

[28] Inker LA (2011). Expressing the CKD-EPI (chronic kidney disease epidemiology collaboration) cystatin C equations for estimating GFR with standardized serum cystatin C values. Am. J. Kidney Dis..

[29] Textor J, van der Zander B, Gilthorpe MS, Liskiewicz M, Ellison GT (2016). Robust causal inference using directed acyclic graphs: The R package ‘dagitty’. Int. J. Epidemiol..

[30] Wilson ME, Boumaza I, Lacomis D, Bowser R (2010). Cystatin C: A candidate biomarker for amyotrophic lateral sclerosis. PLoS ONE.

[31] Tsuji-Akimoto S, Yabe I, Niino M, Kikuchi S, Sasaki H (2009). Cystatin C in cerebrospinal fluid as a biomarker of ALS. Neurosci. Lett..

[32] Ranganathan S (2005). Proteomic profiling of cerebrospinal fluid identifies biomarkers for amyotrophic lateral sclerosis. J. Neurochem..

[33] Ren Y (2015). Measurement of cystatin C levels in the cerebrospinal fluid of patients with amyotrophic lateral sclerosis. Int. J. Clin. Exp. Pathol..

[34] Wilson ME, Boumaza I, Bowser R (2013). Measurement of cystatin C functional activity in the cerebrospinal fluid of amyotrophic lateral sclerosis and control subjects. Fluids Barriers CNS.

[35] Zhu Y (2018). Aberrant levels of cystatin C in amyotrophic lateral sclerosis: A systematic review and meta analysis. Int. J. Biol. Sci..

[36] Watanabe S, Komine O, Endo F, Wakasugi K, Yamanaka K (2018). Intracerebroventricular administration of Cystatin C ameliorates disease in SOD1-linked amyotrophic lateral sclerosis mice. J. Neurochem..

[37] van Eijk RPA (2018). Monitoring disease progression with plasma creatinine in amyotrophic lateral sclerosis clinical trials. J. Neurol. Neurosurg. Psychiatry.

[38] Rosenbohm A (2021). Life course of physical activity and risk and prognosis of amyotrophic lateral sclerosis in a German ALS registry. Neurology.

