# Supplementary material for: Cystatin C based estimation of chronic kidney disease and amyotrophic lateral sclerosis in the ALS registry Swabia: associated risk and prognostic value
Source: Sci Rep. 2023 Nov 10;13:19594. doi: 10.1038/s41598-023-46179-9 (PMC10638424; doi:10.1038/s41598-023-46179-9)
Supplement: Supplementary file 1 — Supplementary Information 1. [file 41598_2023_46179_MOESM1_ESM.docx]

**Supplement**

**Table S1.** Odds ratios for ALS associated with eGFR/CKD-Stages (alternative minimal sufficient adjustment set including cardiovascular disease instead of smoking (ever)).

|  |  | **eGFR/CKD-Stages**  **Odds ratio (95%-CI)** |
| --- | --- | --- |
| **eGFR** | |  |
| **Adjusted c** (N_Cases_=353, N_Controls_=665)^a^ | |  |
| Per 10 units increase | | 0.88 (0.81, 0.96) |
| p-value | | 0.003 |
|  | |  |
| **CKD Stage ≥3** (eGFR <60) | |  |
| **Adjusted c** (N_Cases_=353, N_Controls_=665)^a^ | |  |
| CKD-Stage 1 & 2 | | (ref.) 1.00 |
| CKD-Stage ≥3 | | 1.27 (0.81, 1.99) |
| p-value | | 0.31 |
|  | |  |
| **CKD-Stages** | |  |
| **Adjusted c** (N_Cases_=353, N_Controls_=665)^a^ | |  |
| CKD-Stage 1 | | (ref.) 1.00 |
| CKD-Stage 2 | | 1.74 (1.26, 2.40) |
| CKD-Stage 3 | | 2.07 (1.22, 3.49) |
| CKD-Stage 4 | | 1.37 (0.36, 5.28) |
| CKD-Stage 5 | | - |
| p-value for trend^b^ | | 0.0099 |

^a^ conditioned age and sex, and additionally adjusted for BMI, self-reported diabetes, history of cardiovascular diseases

^b^ p-value for trend over stages

*eGFR estimated glomerular filtration rate, per ml/min/1.73 m^2^; CKD Chronic kidney disease*

**Table S2.** Hazard ratios by eGFR/CKD-Stages among ALS cases (n=362).

|  | **Hazard ratio (95%-CI)** |
| --- | --- |
| **eGFR** |  |
| **Adjusted** **d** (N_Deceased_=303, N_Survived_=44)^a^ |  |
| Per 10 units increase | 1.04 (0.96, 1.13) |
| p-value | 0.29 |
|  |  |
| **CKD-Stages** |  |
| **Adjusted** **d** (N_Deceased_=303, N_Survived_=44)^a^ |  |
| CKD-Stage 1 | (ref.) 1.00 |
| CKD-Stage 2 | 0.79 (0.60, 1.04) |
| CKD-Stage 3 | 0.57 (0.42, 1.08) |
| CKD-Stage 4 | 1.53 (0.54, 4.34) |
| CKD-Stage 5 | - |
| p-value for trend^b^ | 0.16 |

^a^ adjusted for age, sex, diagnostic delay, site of onset, and ALS-FRS, body mass index, self-reported diabetes, smoking (ever), and history of cardiovascular diseases (*history of either hypertension or/and, coronary heart disease or/and, myocardial infarction or/and, stroke).*

^b^ p-value for trend over stages

*eGFR estimated glomerular filtration rate, per ml/min/1.73 m^2^; CKD Chronic kidney disease*

**Figure S1.** Directed Acyclic Graph used for selection of the adjustment variables.
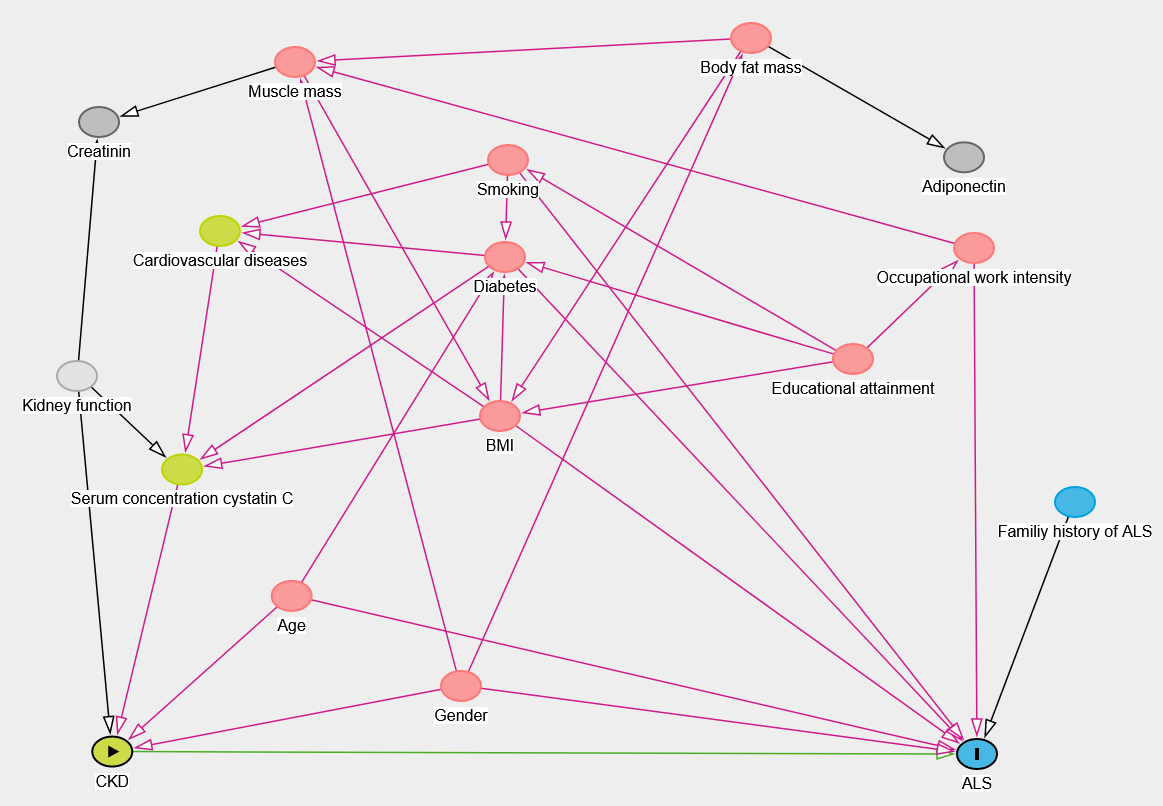


*eGFR estimated glomerular filtration rate, per ml/min/1.73 m^2^; CKD Chronic kidney disease, BMI body mass index*
