# Supplementary material for: Cystatin C based estimation of chronic kidney disease and amyotrophic lateral sclerosis in the ALS registry Swabia: associated risk and prognostic value
Source: Sci Rep. 2023 Nov 10;13:19594. doi: 10.1038/s41598-023-46179-9 (PMC10638424; doi:10.1038/s41598-023-46179-9)
Supplement: Supplementary file 2 — Supplementary Information 2. [file 41598_2023_46179_MOESM2_ESM.docx]

ALS registry Schwaben study group (alle seit 2010):

Alber, B., Klinikum Guenzburg, Department of Neurology

Andres F., Kreiskliniken Reutlingen, Department of Neurology

Arnold G., Klinikum Sindelfingen-Boeblingen, Department of Neurology

Asshauer I., Klinikum Friedrichshafen, Department of Psychiatry and Psychotherapy

Bäzner H., Klinikum Stuttgart, Department of Neurology

Baier H., ZFP Südwürttemberg Weissenau, Department of Epileptology

Baumgärtner J., BKH Augsburg, Department of Psychiatry

Beattie J., Ostalb-Klinikum Aalen, Department of Neurology

Becker T., Ulm University, Klinikum Günzburg, Department of Psychiatry and Psychotherapy II

Behne F., ZFP Südwürttemberg Weissenau, Department of Epileptology

Bengel D., Oberschwabenklinik Ravensburg, Department of Neurology

Bergmeier Christoph Kliniken Landkreis Heidenheim Department of Neurology

Boertlein A., Klinikum Stuttgart, Department of Neurology

Born Ch., Psychiatrie Schwäbisch Hall & PMU Nürnberg, Department of Psychiatry

Bracknies, V., Dietenbronn, Department of Neurology,

Broer R., Klinikum am Weissenhof, Weinsberg, Department of Psychiatry and Psychotherapy

Bürgy M., Klinikum Stuttgart, Department of Psychiatry

Buttmann M., Caritas Krankenhaus, Bad Mergentheim, Department of Neurology

Clauer-Bredt M., Christophsbad Goeppingen, Department of Neurology

Connemann B., Ulm University, Department of Psychiatry and Psychotherapy III

Dempewolf S., RKH Klinikum Ludwigsburg, Department of Neurology,

Demuth Kl., Vinzenz von Paul Hospital Rottweil, Department of Neurology

Dettmers C., Schmieder Kliniken Konstanz, Department of Neurology

Dieterich M., LMU München, Department of Neurology

Etzersdorfer E., Furtbachkrankenhaus Stuttgart, Department of Psychiatry and Psychotherapy

Förch Ch., RKH Klinikum Ludwigsburg Department of Neurology

Freund W., private practice, Biberach

Friederich H., ZfP Zwiefalten, Departmement of Geriatric Psychiatry

Gahr M., Ulm University, Department of Psychiatry III

Gasser Th., University hospital Tuebingen, Department of Neurology

Gebhardt J., ZfP Wiesloch, Department of Geriatric Psychiatry

Gecui I., BKH Memmingen, Department of Psychiatry

Gersner T., ZfP Zwiefalten, Department of Psychiatry and Psychotherapy

Geser F., Christophsbad Göppingen, Department of Psychiatry

Gogolkiewicz A. ZfP Zwiefalten, Departement of Psychiatry

Gold H.-J., Klinikum am Gesundbrunnen Heilbronn, Department of Neurology

Greber R., Vinzenz v. Paul Hospital Rottweil, Department of Geriatric Psychiatry

Grunze H., Psychiatrie Schwäbisch Hall & PMU Nürnberg, Department of Psychiatry

Hacke, W., University of Heidelberg, Department of Neurology

Hamann G., Klinikum Günzburg, Department of Neurology

Hecht M., Kliniken Ostallgäu-Kaufbeuren, Department of Neurology

Heimbach B., University of Freiburg, Department of Neurology

Hemmer B., TU Muenchen, Department of Neurology

Hendrich C., Klinikum Friedrichshafen, Department of Neurology

Henkel K., Christophsbad-Göppingen, Department of Geriatric-Psychiatry

Herting B., Diakonie-Klinikum Schwäbisch Hall, Department of Neurology

Hewer W., Christophsbad Göppingen, Department of Geriatric-Psychiatry

Höglinger G., TU Muenchen, Department of Neurology

Huber R., Klinikum Friedrichshafen, Department of Neurology

Huber-Hartmann K., Kliniken Landkreis Heidenheim, Department of Neurology

Huelser P.-J., Fachklinik Wangen, Department of Neurology

Jöbges M., Schmieder Kliniken Konstanz, Department of Neurology

Jonuz A., Klinikum Schloss Winnenden, Department of Geriatric Psychiatry

Joos A., Kliniken Schmieder Gailingen, Department of Psychotherapeutic Neurology

Jüttler E., Ostalb-Klinikum Aalen, Department of Neurology

Kammerer-Ciernioch J., Klinikum am Weissenhof, Weinsberg, Department of Psychiatry and Psychotherapy

Kaspar A., Oberschwabenklinik Ravensburg, Department of Neurology

Kern R., Klinikum Kempten, Department of Neurology

Kimmig H., Kliniken Schwenningen, Department of Neurology

Klebe, S., University of Würzburg, Department of Neurology

Kloetzsch C., Schmieder Kliniken Allensbach, Department of Neurology and Hegau-Bodensee-Klinikum Singen Department of Neurology

Klopstock Th., LMU München, Department of Neurology

Köhler M., ZfP Zwiefalten, Departement of Geriatric Psychiatry

Kohler A., Klinikum am Gesundbrunnen Heilbronn, Department of Neurology

Kozian R., Vinzenz v. Paul Hospital Rottweil, Department of Geriatric Psychiatry

Kuethmann A., Bezirkskrankenhaus Memmingen, Department of Psychiatry and Psychotherapy

Laske Ch., University hospital Tuebingen, Departement of Geriatric Psychiatry

Lewis D., Marienhospital Stuttgart, Department of Neurology

Lichy C., Klinikum Memmingen, Department of Neurology

Lindner A., Marienhospital Stuttgart, Department of Neurology

Lingor P., TU München, Department of Neurology

Lulé D., Ulm University, Department of Neurology

Maier-Janson W., private practice, Ravensburg

Mäurer M., Caritas Krankenhaus Bad Mergentheim, Department of Neurology

Metrikat J., Bundeswehrkrankenhaus Ulm, Department of Neurology

Meudt O., Klinikum Memmingen, Department of Neurology

Meyer A., Weissenau, Department of Neurology

Michaelides A., Furtenbachkrankenhaus Stuttgart, Department of Psychiatry

Müller vom Hagen J., University of Tuebingen, Department of Neurology

Munk M., University of Tuebingen, Department of Geriatric Psychiatry

Naegele A., Christophsbad Goeppingen, Department of Neurology

Naumann M., University Augsburg, Department of Neurology and Neurophysiology

Neher K.-D., Vinzenz von Paul Hospital, Rottweil, Department of Neurology

Neuhaus O., Kliniken Landkreis Sigmaringen, Department of Neurology

Neusch C., private practice EMSA, Singen

Niehaus L., Klinikum Winnenden, Department of Neurology,

Niestroj A., ZfP Wiesloch, Departement of Geriatric Psychiatry

Opherk C., Klinikum am Gesundbrunnen Heilbronn, Department of Neurology

Pinkhardt E., Klinikum Kempten, Department of Neurology

Raape J., ZFP Südwürttemberg Weissenau, Department od Neurology

Ratzka P., University Augsburg, Department of Neurology and Neurophysiology

Reinhard M., Kliniken Esslingen, Department of Neurology

Rettenmayr C., Klinikum Esslingen, Department of Neurology

Riepe MW., Klinikum Günzburg, Department of Gerontopsychiatry

Rothmeier J., ZFP Südwürttemberg Weissenau, Department of Neurology

Ruchsow M., Christophsbad Göppingen, Department of Psychiatry

Sabolek M., Klinik Biberach, Department of Neurology

Schabet M., RKH Klinikum Ludwigsburg, Department of Neurology

Schaeff-Vogelsang M., Diakonie-Klinikum Schwaebisch Hall, Department of Neurology

Schell C., Kreiskliniken Reutlingen, Department of Neurologie

Schläger A., Kliniken Esslingen, Department of Neurology

Schlipf T., Klinikum Winnenden, Department of Psychiatry and Psychotherapy

Schmauss M., Bezirkskrankenhaus Augsburg, Department of Psychiatry and Psychotherapy

Schoels L., University of Tübingen, Department of Neurology

Schöneberger-Stroick K., BKH Memmingen, Department of Psychiatry

Schörner K., Kliniken Schmieder Gailingen, Department of Psychotherapeutic Neurology

Schuetz K., Kliniken Schwenningen, Department of Neurology

Schweigert B., Caritas Krankenhaus Bad Mergentheim, Department of Neurology

Sheka C., Klinikum Schloss Winnenden, Department of Geriatric Psychiatry

Sommer N., Christophsbad Goeppingen, Department of Neurology

Spannhorst S., Klinikum Stuttgart, Department of Mental Health and Geriatry

Sperber W., Kliniken Esslingen, Department of Neurology

Steber C., Bezirkskrankenhaus Augsburg, Department of Psychiatry and Psychotherapy

Steber R., Bezirkskrankenhaus Memmingen, Department of Psychiatry and Psychotherapy

Stroick M., Klinikum Memmingen, Department of Neurology

Synofzik, M., University Tuebingen, Department of Neurology

Thomas Ch., Klinikum Stuttgart, Department of Mental Health and Geriatry

Trottenberg T., Klinikum Winnenden, Department of Neurology

Tumani H., Ulm University, Department of Neurology

Vasic N., Christiophsbad Göppingen, Department of Psychiatry

Volkmann J., University of Wuerzburg, Department of Neurology

Wahl C., Klinikum Kempten, Department of Neurology

Weber F., Bundeswehrkrankenhaus Ulm, Department of Neurology

Weiler M., University of Heidelberg, Department of Neurology

Weiller C., University of Freiburg, Department of Neurology

Wessig C., University of Würzburg, Department of Neurology

Wick, W., University of Heidelberg, Department of Neurology

Winkler A., TU München, Department of Neurology

Zeller D., University of Würzburg, Department of Neurology
